# The Expression of Leptin and Its Receptor During Tumorigenesis of Diffuse Gliomas such as Astrocytoma and Oligodendroglioma- Grade II, III and IV (NOS)

**DOI:** 10.31557/APJCP.2019.20.2.479

**Published:** 2019

**Authors:** Ramya S Vokuda, B H Srinivas, Venkastesh S Madhugiri, Saravana Kumar Velusamy, Surendra Kumar Verma

**Affiliations:** 1 *Department of Pathology,*; 2 *Department of Neurosurgery, *; 3 *Department of Biostatistics, Jawaharlal Institute of Post Graduate Medical Education and Research (JIPMER), Puducherry, India. *

**Keywords:** Immunohistochemistry, brain tumors, Real-time PCR

## Abstract

**Background:**

Leptin, an adipocytokine functions via the leptin receptor, OB-Rb that contains an intact intracellular domain and activates the JAK/STAT signalling cascade. It stimulates growth, migration and invasion of cancer cells in vitro potentiating angiogenesis. Recently, the involvement of leptin in tumor progression is being explored. Gliomas exhibit poor prognosis, low survival rates demanding for novel therapeutic regimens resulting in discovery of many potential biomarkers and pharmaceutical targets. We analysed the potential role of leptin and OB-Rb in carcinogenesis of malignant gliomas.

**Methods:**

Sixty fresh tissue samples of diffuse gliomas were collected after tumor excision. Real time PCR, immunohistochemical (IHC) analysis and western blot analysis were carried out to assess the expression of leptin and its receptor.

**Results:**

The present study demonstrates the expression of leptin and LepR and their involvement in tumor progression. Of the 60 cases, 57 cases (95%) and 53 cases (88.3%) showed amplification for leptin and OB-Rb respectively. The expression of these proteins were measured semi-quantitatively and correlated with degree of malignancy (p<0.05). The bands were visualised on western blot.

**Conclusion:**

Leptin may be valued as a pharmaceutical target and anti-leptin compounds could be developed as drugs in mono- or combined therapies for these tumors.

## Introduction

Glioblastoma (GBM) is one of the most aggressive brain tumors with high mortality rates (Ohgaki, 2009). Although advanced and specialized treatment modalities exist, the survival rate of patients with GBM remains a concern. This is mainly because glioma cells exhibit resistance to chemotherapeutic drugs and are highly invasive (Rodriguez et al., 2012).

Leptin is one of the cytokines produced by the adipose tissue that is known to be involved in glioma tumorigenesis. However, this has been reported only in in vitro studies on various cancer cell lines (Yeh et al., 2009; Han et al., 2014; Riolfi et al., 2010; Ferla et al., 2011), and thus, in vivo and clinical studies are required to further support this finding. 

Leptin has been shown to potentiate angiogenesis, cell migration, cell survival, and invasion, and inhibit apoptosis in cell lines; these characteristics are yet to be proven in human tumor tissue samples, especially in brain tumors. Leptin can be considered not only as a new biomarker for cancer diagnosis but also as a therapeutic target for personalized treatment regimens. 

The involvement of leptin and its receptor has been widely reported in many cancer types such as breast (Garofalo et al., 2006; Hu et al., 2002; Garofalo et al., 2004; Guo et al., 2012; Ishikawa et al., 2004; Gonzalez et al., 2006), colorectal (Yoon et al., 2004; Koda et al., 2007; Koda et al., 2007; Attoub et al., 2000) prostate (Frankenberry et al., 2004), gastric mucosa (Lee., 2014), esophagal (Beales et al., 2014), ovarian (Kato et., 2015), and endometrial (Sharma et al., 2006; Zhang et al., 2014) cancers. However, except in a few cancer types, such as breast, prostate, and colorectal cancers, the role of leptin in other tumors, such as lung (Unsal et al., 2014) and liver (Saxena et al., 2007) cancer is controversial and warrants further studies to provide a conclusive result. In addition, information on leptin in CNS tumors is limited and very few studies that explain this association exist (Yeh et al., 2009; Han et al., 2014; Riolfi et al., 2010; Ferla et al., 2011; Han et al., 2013; Russo et al., 2004). 

Therefore, in this study, we present leptin positivity in individual grades of diffuse gliomas. Through immunochemistry, western blotting, and real-time PCR, we have shown the expression of leptin and its receptor in various grades of diffuse gliomas.

## Materials and Methods

*Patients*

This prospective study was approved by the Institute Ethics Committee for Human Studies at Jawaharlal Nehru Institute of Post Graduate Medical Education and Research (JIPMER), Puducherry, India. We included all the diffuse gliomas such as Astrocytoma and Oligodendroglioma-Grade II, III and IV (NOS) based on mainly their histomorphological characteristics and a few immunohistochemical markers such as GFAP (Glial Fibrillary Acidic Protein), p53 and Ki-67. The study cohort contained 60 cases [(55% female, 45% male; age range: 18–67 years (mean age = 38.1 years)] of patients with malignant gliomas who underwent surgical resection of the tumor at the Department of Neurosurgery, JIPMER. Informed consent was obtained from all subjects. 

*Immnuohistochemistry*

The expression of leptin and its receptor was evaluated immunohistochemically, as previously described by us (Vokuda et al., 2017), and subsequently confirmed by western blotting. The following primary antibodies were used: Leptin (Ob-A-20) rabbit polyclonal antibody (1:50 dilution; Santa Cruz Biotechnology, Santa Cruz, CA, USA) and Leptin Receptor (ObR-H-300) rabbit polyclonal antibody (1:50 dilution, Santa Cruz Biotechnology). The standardization of these antibodies were done as per manufacturer’s protocol using triple negative grade III breast carcinoma samples. The normal brain tissue were also stained with these antibodies and served as internal controls.

The semi-quantitative scoring system used for assessing the expression of leptin and its receptor is as follows: 

0: negative; +1: < 10% of positive cells (Mild); +2: 10-50% of positive cells (moderate); +3: >50% of positive cells (strong).

**Table 1:** Amplification Protocol

Initial step	Step	Time	Temp
	cDNA synthesis	10 min	50°C
	Taq enzyme activation	15 min	95°C
50 Cycles	Denaturation	20 s	95°C
	Annealing/Data Collection*	20 s	56°C
	Extension	20 s	72°C

* Data collection Leptin receptor gene = FAM channel Internal Control = HEX Channel

**Table 2:** Expression of Leptin Across the Grades of Tumor

Diagnosis	negative	Weak < 10% of tumor cells	Moderate 10-50% of tumor cells	Strong >50% of tumor cells	Total	P value
Grade II	0	8 (53.3%)	6 (40%)	1 (6.7%)	15	
Grade III	0	1 (6.7%)	12 (80%)	2 (13.3%)	15	0.012
Grade IV	3 (10%)	3 (10%)	13 (43.3%)	11 (36.7%)	30	
Total	3 (5%)	12 (20%)	31 (51.7%)	14 (23.3%)	60	

Fisher’s exact test was used to evaluate the association between leptin expression and grade of the tumor

**Figure 1:**
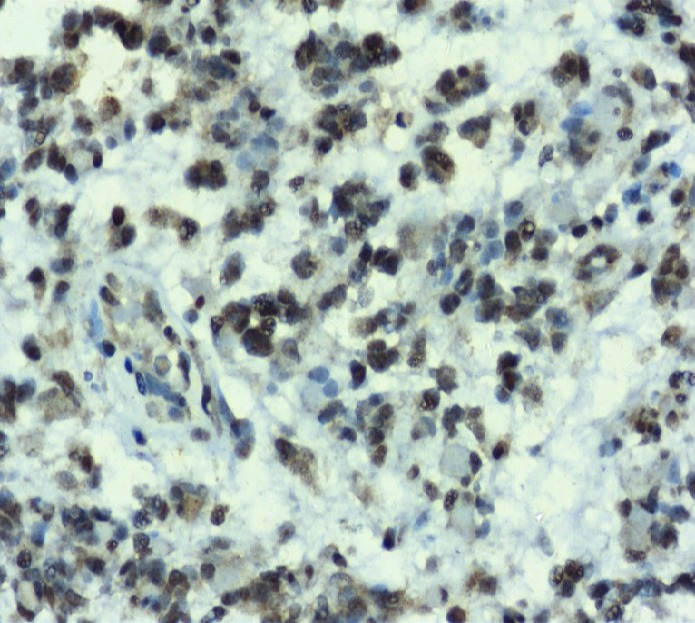
Grade IV – Glioblastoma – x400 - ImmunoHistochemical Expression of Leptin

**Figure 2:**
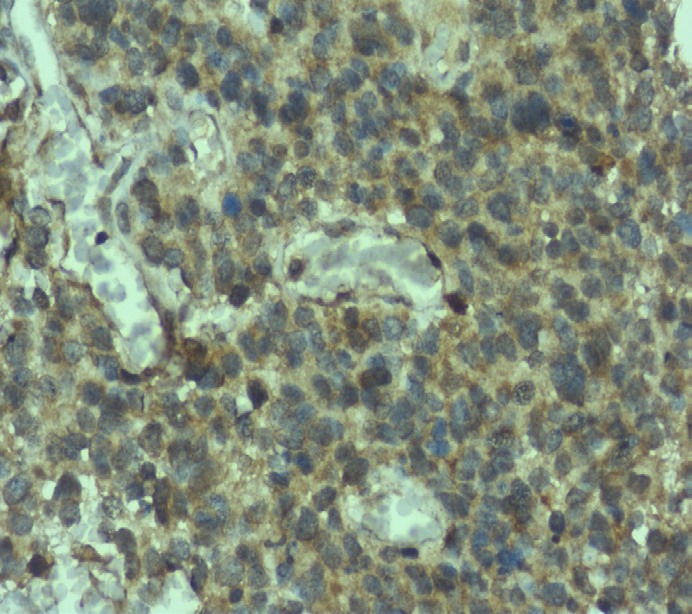
Grade IV- Glioblastoma- x400 - Immunohistochemical Expression of Leptin Receptor

**Figure 3:**
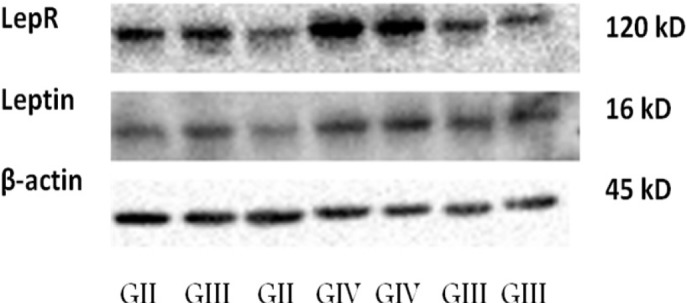
Western Blot Analysis

**Table 3:** Expression of lepR Across the Grades of Tumor

Diagnosis	Negative	Weak < 10% of tumor cells	Moderate 10-50% of tumor cells	Strong >50% of tumor cells	Total	P value
Grade II	0	9 (60%)	6 (40%)	0	15	
Grade III	0	2 (13.3%)	11 (73.3%)	2 (13.3%)	15	0.018
Grade IV	4 (13.3%)	4 (13.3%)	13 (43.3%)	9 (30%)	30	
Total	4 (6.7%)	15 (25%)	30 (50%)	11 (18.3%)	60	

Fisher’s exact test was used to evaluate the association between leptin receptor expression and grade of the tumor

**Figure 4:**
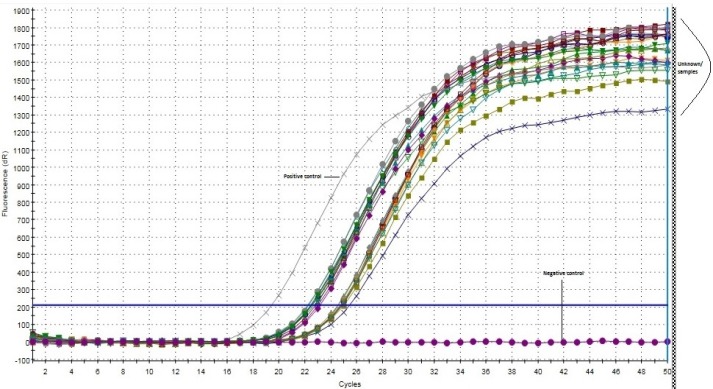
Amplification Plot of Leptin - Real-Time PCR

**Figure 5:**
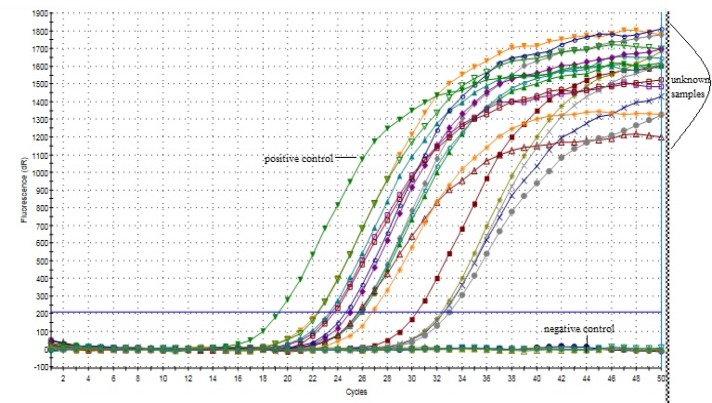
Amplification Plot of Leptin Receptor– Real-Time PCR

*Western blotting*

The tissues samples collected from the study subjects were snap-frozen immediately or immersed in RIPA buffer containing protease inhibitor cocktail (Roche, Basel, Switzerland, Complete Mini) and stored at -80°C till analysis. The samples were prepared in a dounce homogeniser with RIPA buffer (Tris HCL, pH 8, 50 mM; NaCl 150 mM; Nonidet P-40 1%; Sodium deoxycholate 0.5%; Sodium dodecyl sulfate 0.1%) and protease inhibitor cocktail and phosphatase inhibitors (Roche, Indianapolis, IN, USA). The homogenates were centrifuged at 12,000 g at 4ᵒC for 20 min. The supernatant was used to measure the protein content by Lowry’s method. The samples were then treated with sample buffer (5x) at 100°C for 5 min to denature the proteins. Then, the samples (20 µg/well) were subjected to SDS-PAGE (15%). The gel was then transferred to PVDF, and probed with appropriate primary antibodies (mentioned above) overnight. Then secondary antibody tagged with HRP conjugate was incubated for 1 h. The intensity of the band was quantified using ECL reagent (Clarity western ECL kit, Bio-Rad) under Chemi-documentation system (Bio-Rad XRS).

*Real-time PCR*

Gene expression of leptin and its receptor was analyzed using real-time PCR. Normal brain tissue from deceased patients was used as the control. 

*Total RNA purification*

The biopsy tissue was soaked in RNA LATER solution and stored at -80°C until use. The frozen sample were thawed, 50 mg of the tissue was transferred into a fresh 2 ml centrifuge tube, and allowed to saturate to room temperature for 10 min. This was centrifuged at 10,000 rpm for 5 min and the supernatant was carefully discarded by pipetting, as per the manufacturer’s instructions (Helini Biomolecules, Chennai, India). The Probe PCR Master Mix and Primer Probe for Leptin and Leptin receptor were procured from Helini Biomolecules.

Leptin Forward Primer: 

CCTCTGATACCCAGAGCATTAC 

Reverse Primer: CCTCACCTCCTTCAAACTCTAC 

Probe: FAM- 

TGAGCCAGGTAATGAGGGACTGGA-BHQ1 

Leptin Receptor [Long form] 

Forward Primer: CTACCAATGTAGACACGCTCTT 

Reverse Primer: TGGTGTCTGTTGGGCTTATT 

Probe: FAM- AGTGGGAAGGTCCCTTGTTTCCAG-BHQ1

Negative Control setup 

Included 10 µl of nuclease free water 

Positive Control setup

Included 10 µl of Positive control plasmid [target gene cloned plasmid].

*Statistical analysis*

All the statistical analyses were carried out using SPSS Software version 20 (IBM SPSS Inc., Chicago, IL, USA). The association of the expression of leptin and its receptor with age and sex of the patient and grade of the tumor was assessed using Fisher’s exact test. The concordance between leptin and its receptor was assessed using kappa statistics as previously described (Riolfi et al., 2010). Results with a p-value of <0.05 were considered statistically significant.

## Results

*Distribution of diffuse gliomas across our population*

Of the 60 subjects included in the study, 55% (33/60) were females and 45% (27) were males (age range: 18-67 years; mean age: 38.1 years). According to histomorphology and immunohistochemistry, 15 tumors were diagnosed as grade II (12 diffuse astrocytomas and 3 oligodendrogliomas), 15 were diagnosed as grade III (2 anaplastic astrocytomas and 13 anaplastic oligodendrogliomas), and 30 were diagnosed as grade IV glioblastomas.

*Immunohistochemistry of leptin and its receptor*

The expression of leptin and its receptor was assessed by two experienced pathologists who were blinded. The immunohistochemical expression of leptin across grades of tumor have been tabulated (Table 2). The expression of leptin across different sexes was not statistically significant. We observed moderate expression in most of the tumors in terms of intensity. Strong expression was noted in 23.3% of the cases across all grades while only 5% of the cases showed negative expression. As expected, the grade II tumors exhibited weak positivity in 53.3% of the cases while only one case showed strong positivity for leptin. Grade III gliomas showed moderate leptin expression in most of the cases (80%) while strong expression was noted in only two cases. In case of glioblastomas, not much difference was observed between the cases that showed moderate and strong leptin expression. Immunohistochemically assessed expression was significantly associated with the grade of malignancy. Fisher’s exact test was used to assess this association and the p-value was found to be 0.012 (>0.05), which was statistically significant.

The expression of leptin receptor across grades of the tumor has been tabulated (Table 3). Statistical significance was not noted for the expression of leptin receptor among the different sexes. We observed that 6.66% of the cases showed negative expression, 25% showed weak positivity, 50% showed moderate expression, and 18.33% of the cases showed strong positivity of the receptor across all grades. Interestingly, in grade II tumors, we did not observed negativity or strong positivity. Most of the cases (60%) showed weak positivity for the receptor. In case of grade III tumors, moderate positivity was observed in 73% of the cases. In case of glioblastomas, moderate positivity was observed in 43.33% and strong positivity was observed in 30% of the cases analyzed. Similar to leptin, the association of leptin receptor expression across the grades of the tumor was statistically significant with p value being 0.018, as assessed via Fisher’s exact test. 

The overall concordance between leptin and leptin receptor expression in diffuse gliomas (grade II, grade III, and grade IV) was found to be 97.41% with a kappa coefficient of 0.8214.

The photomicrographical images of immunohistochemical staining with leptin and leptin receptor antibodies for glioblastoma are shown in Figures 1-2.

*Western blot analysis*

The results of immunohistochemical analyses were further confirmed by western blot analysis. The expression of leptin and its receptor correlated with the immunohistochemical expression of these proteins in diffuse gliomas. Leptin showed a distinct band corresponding to 16 kDa and leptin receptor showed a band at 120 kDa (Figure 3).

*Real-time PCR analysis of leptin and its receptor*

The mRNA expression of both the genes were analyzed using real-time PCR. The amplification plots have been shown in Figure 4 and Figure 5. The mean Ct* value of leptin and its receptor was found to be 23.86 and 28.27, respectively. 

Of the 60 cases analyzed, 57 (95%) showed amplification for leptin and 3 cases were negative (no amplification). For leptin receptor, 53 (88.3%) cases showed amplification, while 7 cases were negative.

## Discussion

In the current study, we assessed the expression of leptin and its receptor in clinical samples of diffuse gliomas. Although, previous studies have shown the overexpression of these protein in brain tumors, there existed limitations. Riolfi et al., (2010) documented leptin positivity in 55.2% and leptin receptor positivity in 60.9% of cases using immunohistochemistry. They also observed low expression in low-grade astrocytomas and gangliogliomas and increased expression in high-grade astrocytomas. The overall concordance between leptin and its receptor expression was observed to be 80.5%. They noted that the protein expression was statistically significantly associated with the grade of malignancy. Although the positivity of leptin has been reported in general, the intensity of leptin positivity has not been shown in tumor subgroups that were considered for the current study.

In the present study, we were able to detect the expression of leptin and its receptor in a considerably large sample that mostly contained malignant and high-grade tumors. Leptin and its receptor were cytoplasmically expressed in tumor cells and were negative in normal astrocytes as observed in other studies (Riolfi et al., 2010; Garofalo et al., 2006; Ishikawa et al., 2004; Koda et al., 2007; Koda et al., 2007; Koda et al., 2007). 

In real-time PCR analysis, we observed leptin mRNA expression in 95% of the cases. The cases that showed negative expression on IHC showed leptin mRNA amplification in PCR. This may be attributed to sensitivity and specificity of the method. In case of mRNA expression of leptin receptor, we observed amplification in 88.3% of the samples. Ten samples showed high Ct* value (>30.0) but showed good peak which implies that low copy number of these genes may be present in the sample. These were mostly grade II tumors. 

From these results, the difference in the protein and mRNA expression analyses puts forth the question about epigenetic modifications that may occur with respect to these molecules. 

Han et al., (2014) confirmed that leptin plays an active role in promoting human glioblastoma growth via the JAK/STAT signaling pathway. Their experiments were carried out in vitro using U87 human glioblastoma cell lines (Han et al., 2014). A study claimed that leptin receptor-positive glioblastoma cells were resistant to chemotherapy with temazolamide (TMZ) which failed to arrest cell proliferation and initiate apoptosis via STAT3 signaling. This signaling pathway is thought to play a role in self-renewal in leptin receptor-positive cells and TMZ resistance. Significant expression of the leptin receptor has been shown in CD133+ cells and knockdown of leptin receptor effectively lowered the invasiveness of stem-like cells in gliomas and confirmed that JAK/STAT3 signaling is the molecular mechanism involved in the invasion of U87 glioma cells (Han et al., 2013). Another study suggests ObR as an important target for anti-cancer therapy as the ObR+ glioblastoma cells possessing glioblastoma stem cell properties were associated with vasculogenic mimicry formation and microvascular density (MVD) (Han et al., 2017). Extensive research has been carried out in mammary tumors with respect to these antibodies. Leptin and its receptor have been shown to be positively expressed in primary breast tumors and lymph node metastasis via IHC7. A positive correlation of leptin and its receptor has been observed, which was also statistically significant with a concordance of 93% between the two markers. Inhibition of leptin signaling has effectively reduced the tumor activity in breast carcinomas15. Similar effects of leptin and OB-R has been reported in colorectal carcinoma (Koda et al., 2007; Koda et al., 2007), papillary thyroid carcinoma30, and gastric carcinomas (Lee et al., 2014). In endometrial tumor, the expression of leptin and OB-R was also positively associated with nodal metastasis, the intensity of invasion of the myometrial tissue, and poor prognosis (Zhang et al., 2014: Koda et al., 2007).

The real-time PCR analysis of expression of leptin and its receptor has been carried out so far only in rat prostrate, which revealed the amplification curves of leptin cDNA alone (Malendowicz et al., 2006). In another study on pituitary adenomas, intact Ob-Rb was documented in 89.6% of the cases by RT-PCR (Jin et al., 1999). We conducted real-time PCR analysis for expression of both the genes. The amplification plots and their respective mean Ct*values confirm the involvement of these genes in tumor progression. 

High mRNA expression of leptin (99%) and leptin receptor (100%) has been noted in breast cancer10. As compared with the other studies, our study exhibits the highest positivity of these molecules using IHC and real-time PCR which may be attributed to the samples selected for the study that mainly involve grade IV tumors followed by grade III and II. The bands of the respective proteins as observed on western blot are also in line with the assays conducted by other authors (Attoub et al., 2000: Cheng et al., 2011: Oliveira et al., 2001). 

The use of antagonists against leptin receptor, leptin, or the signaling pathway (JAK/STAT) may prove promising in the treatment of brain tumor. Various inhibitors and antagonists are being developed in this regard.

However, there are no clinical trials on these antagonists and inhibitors. They have been experimented only on cells lines and in animal models (Gonzalez et al., 2006; Frankenberry et al., 2004; Lee et al., 2014; Sharma et al., 2006; Saxena et al., 2007; Cheng et al. 2011; Gonzalez et al., 2009).Targeted therapeutic strategies combined with anti-leptin compounds may have the potential to increase and improve the efficacy of cancer therapy protocols. 

The current study provides initial insights toward the understanding of the probable involvement of leptin and its receptor in diffuse glioma tumorigenesis, indicating that the leptin system may be involved in glioma pathogenesis through autocrine or paracrine mechanisms. The expression of leptin and its receptor signals via the JAK/STAT pathway to bring about its neoplastic action in these tumors has been positively associated with the degree of malignancy. Leptin signaling and cross-talk with other molecular pathways play a critical role in angiogenesis, proliferation, invasion, and migration of diffuse glioma cells. Inhibition of leptin action using specific antagonists and inhibitors against specific signaling pathways might prove promising for the treatment of gliomas and effective management in these patients with glioma to increase their quality of life. 

This study has a few limitations. The difference of expression in astrocytomas and oligodendrogliomas across respective grades have not been analysed due to lesser sample size. The study focuses on correlation of leptin expression across various grades considering diffuse gliomas as a single cohort. The follow-up analysis on these patients has not been conducted. Therefore, the prognostic value of these markers on diffuse gliomas is inconclusive. In addition, in vitro experiments using inhibitors and antagonists have not been carried out to deduce the exact molecular mechanism of leptin in these tumors. The regulation of leptin expression via gene mutations or epigenetic mechanisms are yet to be explored. Further, the interactions between leptin and other signaling pathways, such RTK signaling, that have been implicated in gliomas are yet to be analyzed. 

## Statement conflict of interest

None.
